# Active Drumming Experience Increases Infants’ Sensitivity to Audiovisual Synchrony during Observed Drumming Actions

**DOI:** 10.1371/journal.pone.0130960

**Published:** 2015-06-25

**Authors:** Sarah A. Gerson, Andrea Schiavio, Renee Timmers, Sabine Hunnius

**Affiliations:** 1 University of St Andrews, School of Psychology & Neuroscience, St Andrews, United Kingdom; 2 Donders Institute for Brain, Cognition, and Behaviour, Center for Cognition, Radboud University, Nijmegen, The Netherlands; 3 Music Mind Machine in Sheffield, Department of Music, The University of Sheffield, Sheffield, United Kingdom; Eberhard Karls University of Tuebingen Medical School, GERMANY

## Abstract

In the current study, we examined the role of active experience on sensitivity to multisensory synchrony in six-month-old infants in a musical context. In the first of two experiments, we trained infants to produce a novel multimodal effect (i.e., a drum beat) and assessed the effects of this training, relative to no training, on their later perception of the synchrony between audio and visual presentation of the drumming action. In a second experiment, we then contrasted this active experience with the observation of drumming in order to test whether observation of the audiovisual effect was as effective for sensitivity to multimodal synchrony as active experience. Our results indicated that active experience provided a unique benefit above and beyond observational experience, providing insights on the embodied roots of (early) music perception and cognition.

## Introduction

Sensitivity to the integration of information across multiple senses is crucial to perceiving and acting in a multimodal environment. In this paper, we focus on a classic question in the field of development: *when* and *how* do infants gain sensitivity to synchrony between visual and auditory modalities? A variety of research suggests that infants are able to recognize synchrony and violations of synchrony between visual and auditory modalities in some contexts. On a broad level, infants as young as four months recognize that visual presentations are associated with particular sounds. For example, they are more likely to look to a person covering her eyes than a musical instrument when hearing “peek-a-boo” [1]. On a more fine-grained level, infants also differentiate between synchronous and asynchronous presentation of auditory and visual stimuli [2–4]. The threshold at which individuals discriminate synchronous and asynchronous presentation of stimuli, however, changes over development (see [5–6]) such that infants and children become increasingly sensitive to shorter offsets between visual and auditory stimuli over time [5,7]. For example, although six-month-old infants did not behaviourally demonstrate sensitivity to a 200 ms offset between auditory and visual presentation of stimuli, they showed sensitivity to this offset via event-related potential components measured with electroencephalography during perception of synchronous versus asynchronous stimuli [8]. At ten months of age, when habituated to a synchronous audiovisual event, infants detected asynchrony when the offset between visual and auditory information was 666 ms, but not when the offset was 366 or 500 ms [6].

Development of sensitivity to multisensory synchrony has several plausible mechanisms, including brain maturation, visual experience, and multisensory experience. Most researchers are in agreement that a combination of maturation and different types of experience contribute to this development (e.g. [9–10]). Lewkowicz [11] claims that “various findings on the effects of early experience demonstrate unequivocally that the young nervous system is highly plastic and that it depends on exposure to temporally and spatially aligned multisensory inputs for the development of normal multisensory functions” (p. 6). This position is supported by a variety of studies (in both animal models and special populations, such as children born deaf and later receiving cochlear implants) suggesting that limited input from one of the senses early in development limits sensitivity to multisensory integration even when that sense is later recovered (e.g. [12]). Although the importance of general experience has been emphasized in this domain, the relevance and contribution of particular types of experiences still need to be thoroughly examined in order to better understand the differential role of different kinds of experience.

The everyday life of an infant consists of a variety of activities that engage multiple modalities simultaneously. For example, infants’ manual explorations [13], affective tuning [14], action perception [15], motor planning [16], language and memory development [17–18], and music-related behaviors [19–20], all have cross-modal components that could play a key role in developing multimodal sensitivities. Sheya and Smith [16] suggested that these various forms of multimodal experiences are a “core mechanism creating developmental change” in that they influence the ordering of development and the development of higher-order cognition (p. 125). The active creation of multimodal experiences by the child (i.e., active experience) and the development in a variety of domains that ensues thus interact and create a cyclical perpetuation of change that links multimodal experience and cognitive development bidirectionally [21–22]. The study of sensitivity to multisensory synchrony in human development, therefore, cannot be isolated from the dynamics of the experience the child encounters and creates in daily life.

In light of these considerations, musical contexts provide rich opportunities to investigate this development, as they consist of an intrinsic interplay between action, multimodal activities, and cognition [23–25]. The present study thus helps to forge the link between research on musical development, sensitivity to multisensory synchrony, and perception-action coupling in infancy. Given this background, our aim is to investigate the role of active experience (i.e., motor experience with an action that results in audiovisual effects) on sensitivity to multisensory synchrony (or violations of synchrony) in infants in a musical context.

## Active Experience as a Source of Sensitivity to Multisensory Synchrony

It is perhaps intuitive that experience perceiving the integration of two senses (e.g., hearing and seeing for audiovisual stimuli) might help one recognize the synchrony between visual and auditory events. Less obvious, however, is the unique role active experience may play in this-i.e. creating the co-occurring visual and auditory stimuli. Links between active production of sensory effects and perception of these effects have been found using behavioral and neurophysiological measures across development. For example, three-month-old infants trained to produce object-directed actions using Velcro mittens recognized the causality inherent in the visually perceived consequences of those motor actions, but infants who did not receive active training did not [26]. In a study assessing the neural effects of multimodal experience [27], infants were trained to use a tool to hit an object that then created a sound, or they observed their parent create this sound with the same action. Infants later showed more motor activation (as assessed via electroencephalography) to the sound associated with the previously performed action than to the sound associated with the observed action.

Similar effects of motor expertise have been found in adults. Behavioral research with adult experts reveals that individuals with skilled motor expertise (e.g., professional basketball players) are more accurate at predicting the timing of others’ actions than individuals with visual, but not motor, experience (e.g., basketball coaches; [28]). Relatedly, professional dancers show more motor activity when viewing actions within their motor repertoire than those with which they have similar amounts of observational experience but cannot perform themselves [29].

These findings suggest that active experience uniquely influences the perception and prediction of others’ actions. The effect of experience on sensitivity to multisensory synchrony relates closely to these results, particularly within musical contexts. As reviewed by Paraskevopoulos and Herholz [30], a range of research indicates that experience as a musician is related to altered perception of audiovisual integration and related changes in brain structure and function. For example, piano players are more sensitive than novices to audiovisual asynchrony of piano playing, but not of speech [31] and drummers are more sensitive than novices to audiovisual asynchrony of drumming actions (e.g. [32–33]). In addition to long-term effects of musical training on sensitivity to audiovisual integration, experimental manipulations using short-term musical training have provided more direct evidence that sensorimotor-auditory training creates behavioral and brain changes (e.g., [34]).

Although passive perception of multisensory events contains the information necessary for recognizing integration of stimuli across the senses, research directly contrasting active and passive (i.e., observational) experience with multisensory stimuli indicates that active engagement plays a unique role. For example, Butler and colleagues [35–36] found that adults were faster and more accurate at recognizing audiovisual associations learned through active manipulation of an object with which these associations were paired than through passive visual learning of the associations. Developmental research assessing the effects of active versus passive experience on multisensory integration is relatively rare. An exception to this is a study by James and Bose [37] in which 5- to 7-year-old children received active or passive training with objects that were paired with sounds and then heard the sounds associated with the actively or passively learned object-sound pairing. In this study, there was greater motor cortex activation for the sounds learned in the active condition than in the passive condition, suggesting a specific effect of active experience on multisensory integration at the neural level.

Despite the fact that some research has addressed the role of active and passive training of multisensory actions in infants, this work has largely focused on outcomes within only one sensory system (i.e., auditory domain). For example, Gerry and colleagues [38] found that 12-month-old infants who had received six months of experience in an interactive musical setting were more likely than their peers (who received passive experience with music) to prefer tonality that was matched to Western norms. Similarly, Phillips-Silver and colleagues [39] either bounced infants on an experimenter’s knee to certain beats or had the infants observe an experimenter bounce her knee (without being engaged in the movement with the experimenter) and found that the experience of bouncing influenced infants’ perception of the beat, whereas observation had no such effect. These findings suggest that manipulating infants’ active versus passive experience with music plays an important role in infants’ auditory perception. Investigating the effects of training experience on sensitivity to multisensory synchrony in infants will provide insight into the intricate relations between the developmental origins of multisensory experiences, perception, and learning. Extending previous findings, we predict that active experience is particularly important to the developing sensitivity to multisensory synchrony of (matched) actions in infants.

In the present research, we altered infants’ experience with multimodal actions and assessed the effects of this manipulation on their sensitivity to multisensory synchrony. At six months of age, infants’ perception of synchrony in multimodally presented stimuli is variable depending on the offset in presentation of different modalities [6, 8]. We hypothesized that training young infants to produce novel multisensory drumming actions would facilitate their ability to recognize the synchrony of the multisensory consequences of these same actions. To test this hypothesis, we gave six-month-old infants active or passive experience with the production of drumbeats and compared their perception of drumming synchrony versus asynchrony in this condition relative to a condition in which they received no such experience. In a second experiment, we then contrasted this active experience with observational experience of drumming in order to assess whether observation of the audiovisual effect was as effective as active experience. Given the unique effects of active versus observational experience on adults’ perception of synchronous versus asynchronous audiovisual stimuli *and* the unique effect of active versus observational experience on infants’ perception of others’ actions, we expected that active experience would uniquely influence multisensory perception (i.e., detection of synchronous versus asynchronous audiovisual stimuli) in infants.

## Experiment 1

### Methods

#### Participants

Forty six-month-old infants (range: 5 months, 16 days to 7 months, 0 days) participated in one of two conditions: active training (n = 20, *M* age = 6 months, 17 days) or control (n = 20, *M* age = 6 months, 13 days). Infants were recruited from a database of families interested in participating in infant research. Before testing began, all parents signed a written informed consent that explained the procedure and noted that they could discontinue participation at any point for any reason. Some parents (including the participants pictured in figures) also gave written permission for anonymous photos and videos to be shared in academic publications or lectures. The ethics committee associated with research at our institute (facultaire Ethische Commissie Gedrags-wetenschappelijk onderzoek [ECG]) approved this study (approval number: ECG2012-1301-006).

#### Procedure

Infants in both conditions had the chance to play with a drumming toy for approximately five minutes. In the active training condition, infants interacted with the drum prior to watching the video stimuli. In the control condition, infants interacted with the drum after watching the video stimuli.

**Fig 1:**
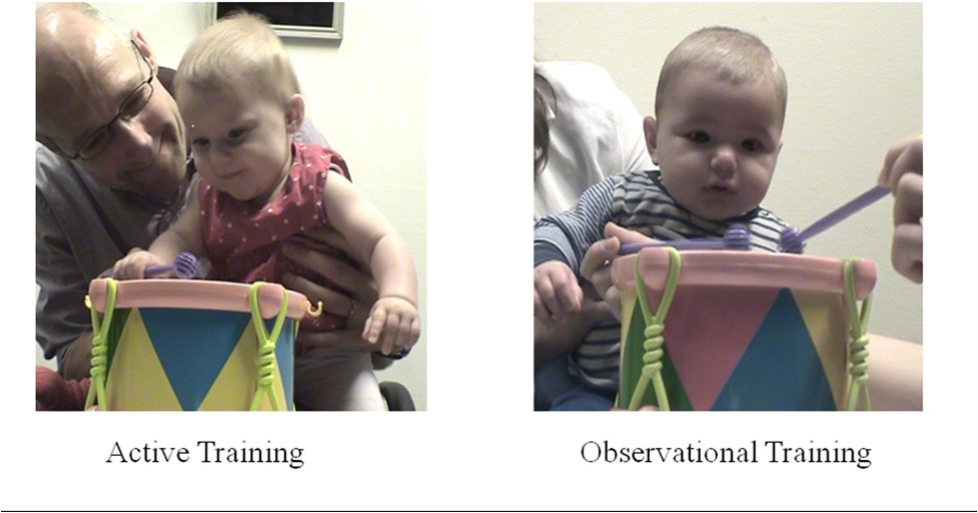
Training Conditions. Infants in the active condition played with drum and infants in the observational condition observed drumming for approximately five minutes prior to watching the films.

During training, the infant sat on a parent’s lap and a table was placed in front of them with a small drum and two drumsticks (see **Fig 1**). One of the drumsticks was placed in the infant’s hand and the experimenter drew the infants’ attention to the drum by tapping and gazing toward the drum. If the infant did not make contact with the drum using the drumstick, the experimenter demonstrated the drumming action with the other drumstick.

**Fig 2:**
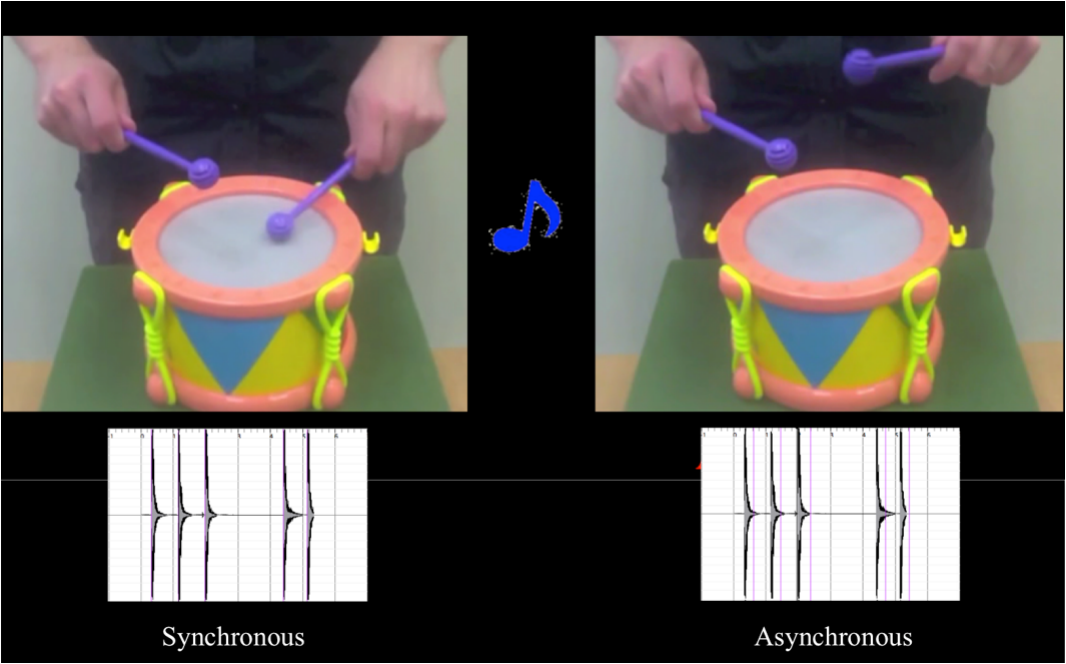
Video Session. Infants saw two simultaneously presented movies, one of which was synchronous with the audio and the other was offset by 300 or 600 ms. The visual representations of the sound-waves for each video are displayed below the videos (with “visual beat” represented by purple lines).

Either before (control condition) or after (active training condition) training, infants saw a series of eight video pair trials. The infants saw two videos presented next to one another (see **Fig 2**). The videos started simultaneously and were identical except that the two video displays were offset from one another by 300 or 600 ms. In all videos, an actor beat a drum to the same musical beat for thirty seconds. The actor’s face was not visible and the only sound was that of the drumstick hitting the drum. The audio of one of the two videos was presented from a neutral location behind the screen. In this way, the drumming sound was synchronous with one of the two videos and either 300 or 600 ms early or late for the other video. Across eight randomly presented trials, infants saw all possible combinations of offset: 300 and 600 ms early and late (audio relative to video). The order of trials and when the synchronous video was on the left or right side of the screen was pseudorandomized (with the requirement that half of the synchronous videos were presented on each side across trials) and varied between subjects. Between each video, a short attention getter (a sound and an object, but no action that could be considered synchronous or asynchronous with the sound) was presented in the center of the screen to direct the infant’s attention back to the video if he or she had become distracted.

#### Coding of behavior

All sessions were recorded via a video camera facing the infant, directly below the center of the presentation screen. These videos were coded offline to assess infants’ activity during the training sessions and their direction of looking during the video session.

A trained coder watched all training sessions and counted the number of times infants made contact with the drum using the drumstick or his or her hands. The coder also counted the number of times the parent or experimenter made contact with the drum. Additionally, she noted the number of times the infant was not looking during contact, as watching the event that produced the sound would be particularly important for learning multisensory associations. A second coder double-coded 25% of the sessions and the number of hits coded as produced and observed by the two coders was highly correlated (*r*s = .94).

A trained coder, blind to the side of the screen on which the synchronous video was playing, conducted frame-by-frame coding to indicate whether the infant was looking to the left or right side of the screen throughout each of the eight video segments. Following coding, a logfile was consulted to extract information about each segment: on which side the synchronous video was played, the sound offset (300 or 600 ms), and the timing (early or late). Using this information, the amount of time infants spent looking at the synchronous versus asynchronous video across each type of test trial could be assessed. A second coder double-coded 25% of the sessions and the two agreed on whether the infant looked longer to the synchronous or asynchronous video on 93% of trials. Raw looking times for these sessions were highly correlated between coders, *r*s > .90.

### Results

#### Training session results

On average, infants in the active training condition saw themselves make contact with the drum approximately 85 times (SEM = 9.75). In the control condition (during the play session following video session), on average, infants saw themselves make contact with the drum approximately 54 times (SEM = 7.80). A t-test indicated that infants in the active training condition produced (and observed) more drum hits than infants in the control condition, *t*(38) = 2.51, *p* = .016. This was likely a function of fatigue at the end of the testing session for infants in the control condition. In accordance with fatigue over time, infants in the active training condition, who played with the drum before watching the videos, spent less time (overall) watching the videos (M = 69.60 seconds [SEM = 2.66]) than infants in the control condition, who watched the videos at the beginning of the session (M = 81.87 seconds [SEM = 2.91]; *t*(38) = 3.11, *p* = .003).

#### Video session results

Initial analyses examined whether early versus late onset of videos relative to audio (order onset), 300 ms versus 600 ms delays between video and audio (delay offset), or age played any role in infants’ relative attention to the events displayed. No main effects or interactions with order onset, delay offset, or age were found (*p*s > .14, η_p_^2^ < .06). We thus collapsed across these different types of trials and did not include age in subsequent analyses. In order to assess whether infants looked longer to synchronous versus asynchronous videos across trials, we conducted a Repeated Measures Analysis of Variance (ANOVA) with total looking time to synchronous and asynchronous videos as the within-subjects, repeated measures variable and Condition (active training or control) as the between-subjects variable. This revealed a significant interaction between Synchrony and Condition, *F*(1,38) = 4.31, *p* = .045, η_p_^2^ = .10. No main effect of Synchrony was found across conditions, *F*(1,38) = .05, *p =* .82, η_p_^2^ = .001, but a main effect of Condition emerged, *F*(1,38) = 9.70, *p* = .003, η_p_^2^ = .20. The main effect of Condition was consistent with the above-described pattern of infants in the control condition attending to the videos for more time than infants in the active condition. Pairwise comparisons revealed that the interaction between Synchrony and Condition was driven by differences between looking times to the asynchronous event in the active training and control conditions, such that infants in the control condition spent more time (*estimated marginal mean =* 42.25 seconds [*SEM* = 1.79]) attending to the asynchronous events than infants in the active condition (*estimated marginal mean =* 36.68 seconds [*SEM* = 1.83]), *mean difference* = 9.53, *p* = .001. The difference between conditions in attention to the synchronous events was not significant, *mean difference* = 2.74, *p* = .20.

**Fig 3:**
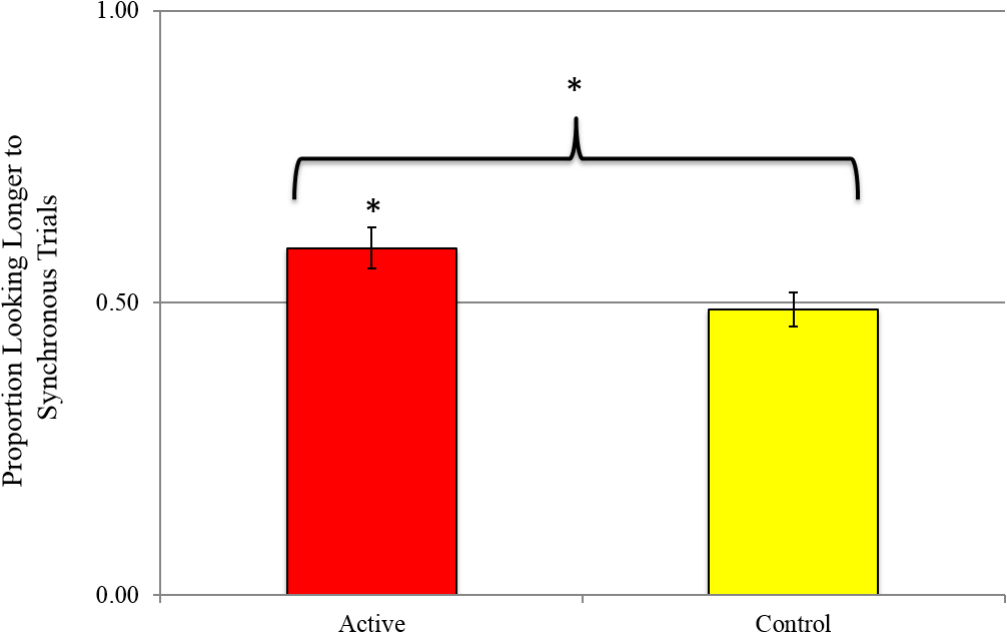
Experiment 1 results. Infants in the Active and Control conditions differed in the proportion of trials in which they looked longer to the synchronous videos. (*p < .05, chance = 50%, error bars represent standard errors).

In order to both control for differences in raw looking times to events and to assess consistency in preference for synchronous or asynchronous videos across trials, we created a score that measured whether infants consistently looked longer to synchronous or asynchronous events across trials by assigning each infant a binary score of 0 for each trial for which he or she looked longer to the asynchronous video and 1 for each trial for which he or she looked longer to the synchronous video. The proportion of the eight trials that each infant looked longer to synchronous videos was then used as a dependent variable. An independent samples t-test indicated that infants in the two conditions differed in the proportion of trials that they looked longer to synchronous videos (see **Fig 3**), *t*(38) = 2.27, *p* = .03 (Cohen’s *d* = .72). In order to follow up on this effect of condition, one-sample t-tests were conducted for each condition to determine whether the proportion of trials for which synchronous videos were preferred differed from chance (50%). In the active training condition, infants looked longer at synchronous videos on approximately 60% of the trials; this differed significantly from chance level: *t*(19) = 2.73, *p* = .01 (Bonferonni corrected; Cohen’s *d* = 1.25). In the control condition, infants looked toward synchronous videos on approximately 49% of trials, which did not differ from chance, *t*(19) = -.29, *p* = .78 (Cohen’s *d* = .13).

Non-parametric binomial tests confirmed these findings. A significantly greater number of infants in the training condition looked longer to synchronous trials than asynchronous trials (sign-test: *p* = .02), but the number of infants who looked longer to these different types of trials did not differ for infants in the control condition (sign-test: *p* = .79). All data reported are available in **S1 File.**

### Discussion

The results of this experiment indicate that experience creating multisensory actions was beneficial for sensitivity to the synchrony of multisensory stimuli. That is, infants who received active training producing multisensory actions preferred to look toward synchronous, relative to asynchronous, presentations of the action they had just learned. In contrast, infants who did not receive training producing the action beforehand showed no such preference. These findings were corroborated by both raw looking times to the events and a measure of consistency across trials that controlled for overall looking time. Specifically, when examining raw looking times, infants in the active and control conditions differed in their relative attention to synchronous and asynchronous events such that infants in the control condition looked more to asynchronous events than did infants in the active condition. Thus, infants in the control condition were attending more to the mismatched event, whereas infants who had received active training did not look as long to these events, presumably because they did not match their expectations. These findings were further supported by analyses that took into account the consistency with which infants looked to synchronous versus asynchronous events across trials. According to this measure, infants in the two conditions differed in their consistent preference for synchronous events, such that infants in the active condition looked to synchronous events across more trials than would be expected by chance, whereas infants in the control condition did not.

These findings are in accord with animal research that indicates a key role for early experience in multisensory integration [12]. Impressively, a mere five minutes of training (similar to active training in other research; e.g., [40–41]) was sufficient to influence six-month-old infants’ differentiation between synchronous and asynchronous audiovisual presentations.

The above findings are important in highlighting the role of experience in the increasing sensitivity to multisensory synchrony. They do not, however, shed light on what particular kinds of experience are most effective in contributing to sensitivity to multisensory synchrony. It is possible that the mere observation of the synchronized audio and visual stimuli is sufficient to influence perception of multisensory integration. In contrast to this hypothesis, research discussed above (e.g. [37, 40]) points to the possibility that active experience producing the action (i.e., engaging one’s body and motor system during the perception of the sensory consequences) might be particularly effective in altering multisensory perception. Both behavioral and neural research with adults and children has shown unique effects of active, above and beyond observational, experience on multisensory integration (e.g. [37]). In infancy, unique effects of active movement have been found for action understanding (e.g. [42–43]) and for musical preferences (e.g. [39]). In order to uncover whether observational experience with multisensory actions is sufficient to induce change in sensitivity to the synchrony of multisensory stimuli, it is necessary to assess this sensitivity in infants who receive observational training with the same actions as those produced in Experiment 1. In Experiment 2, we assess the effect of a matched amount of observational experience of drumming on sensitivity to multisensory synchronization.

## Experiment 2

### Methods

#### Participants

A separate set of twenty six-month-old infants (range: 6 months, 1 day to 6 months, 27 days) participated in Experiment 2 (*M* age = 6 months, 12 days). The age of infants in Experiment 2 did not differ from infants in either condition in Experiment 1 (one-way ANOVA: *p* = .27; posthoc LSDs comparing to each condition: *ps* > .12). Infants were recruited from a database of families interested in participating in infant research. Before testing began, all parents signed an informed consent that explained the procedure and noted that they could discontinue participation at any point for any reason (see note about ethical approval in Experiment 1).

#### Procedure

Infants first underwent a five-minute observational training session and then observed the same exact video session as infants in Experiment 1. Coding of infants’ behavior was identical across experiments, with the exception that the training session was only coded for parent/experimenter actions and not infant actions (as the infant produced no actions). The observational training session was created to match the types of experience infants in the control condition from Experiment 1 received, as described below. A second coder double-coded 25% of looking times in the video sessions and the two agreed on whether infants looked longer to synchronous or asynchronous videos on 95% of trials. Raw looking times for these sessions were highly correlated between coders, *r*s > .98.

During training, the infant sat on a parent’s lap and a table was placed in front of them with a small drum and two drumsticks (see **Fig 1**). The experimenter then proceeded to produce drumbeats on the drum with the drumstick at a pace comparable to that of infants in the control condition from Experiment 1. If the infant was inattentive, the experimenter tried drawing infants’ attention back to the drum. For one participant, the infant was distracted by the experimenter and the parent was asked to provide drumming examples for the infant. A second coder double-coded 25% of the observational training sessions and the number of hits coded as produced (by the experimenter) and observed (by the infant) by the two coders was highly correlated (*r*s ≥ .99).

### Results

#### Training session results

We first assessed the number of hits produced by the experimenter and the subset observed by the infant during the observational training session in order to confirm that the amount of experience received by infants in this experiment was matched to that of infants in the active training condition from Experiment 1. The number of hits produced by the child or experimenter, respectively, was closely matched in the active and observational training conditions (*M* = 118.5 [*SEM* = 12.04] and *M* = 119.3 [*SEM* = 6.48], respectively; *t*(29.15) = .06, *p* = .96 [correcting for unequal variances]). The number of hits observed by the infants in the two conditions differed in that infants in the observational training condition observed more hits than infants in the active training condition (observation: *M* = 108.75 [*SEM* = 29.04], *t*(38) = .10, *p* = .05). Infants in the observational condition thus received slightly more experience observing the multisensory stimuli prior to the video testing session than did infants in the active training condition from Experiment 1.

#### Video session results

As in Experiment 1, we first examined whether there were effects of onset order, delay offset, or age for raw looking times. No effects of onset order or age were revealed, *p*s > .60, η_p_^2^ < .015. A Repeated-Measures ANOVA with raw looking times as dependent variables and Synchrony (synchronous or asynchronous) and Delay Offset (300 ms or 600 ms) as within-subjects factors revealed a significant interaction between Synchrony and Delay Offset in Experiment 2, *F*(1,19) = 6.16, *p* = .023, η_p_^2^ = .24 and no main effects (*p*s > .60, η_p_^2^s < .015). Follow-up pairwise comparisons, however, revealed no significant differences between looking toward synchronous and asynchronous events at either 300 ms or 600 ms offset (*p*s > .08) or differences between offsets for either synchronous or asynchronous events (*p*s > .14). Binomial tests for each delay offset further indicated that the number of infants who looked longer to synchronous than asynchronous trials did not differ from chance in either delay offset (*p*s > .25). As in Experiment 1, we also assessed consistency in preference for synchronous or asynchronous videos across trials using the proportion measure (proportion of trials for which infants preferred looking to synchronous over asynchronous videos). One-sample t-tests indicated that infants in Experiment 2 did not differentiate between synchronous and asynchronous trials in either 600 ms offset, *t*(19) = -.69, *p* = .50 or 300 ms offset trials, *t*(19) = .78, *p* = .45.

**Fig 4:**
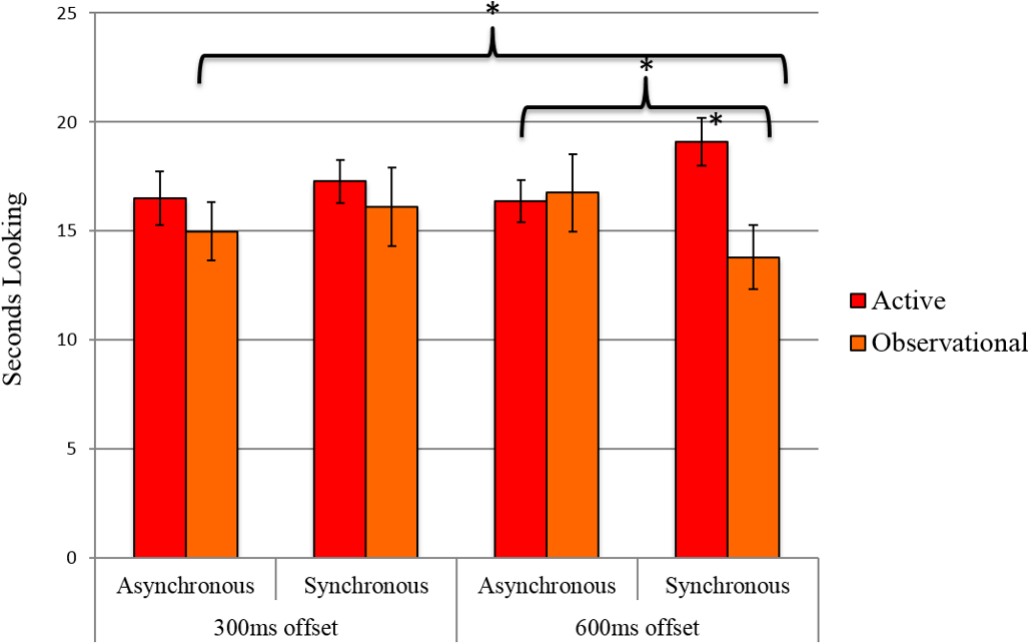
Active and Observational Results. Infants in the Active (Exp 1) and Observational (Exp 2) conditions differed in the amount of time they looked to synchronous videos during 600 ms offset trials. (*p < .05, error bars represent standard errors).

We then conducted a Repeated-Measures ANOVA to directly contrast infants in the active training condition from Experiment 1 with infants in Experiment 2. Given the interaction between Synchrony and Delay Offset in Experiment 2, delay offset was included in the analyses. This ANOVA revealed a significant Synchrony by Delay Offset by Condition interaction, *F*(1,38) = 5.22, *p* = .028, η_p_^2^ = .12, and no other main effects or interactions, *p*s > .17, η_p_^2^ < .05. In order to examine this interaction more closely, separate ANOVAs were conducted for the 300 ms and 600 ms delay offsets. No main effects of or interactions between Synchrony and Condition were found for the 300 ms offset delay, *p*s > .35, η_p_^2^ < .03. Thus, when collapsed across conditions, there is no effect of synchronicity at a 300 ms delay offset, suggesting that infants did not demonstrate sensitivity to synchrony at this offset regardless of training. For the 600 ms delay offset, a significant interaction between Synchrony and Condition was revealed, *F*(1,38) = 6.54, *p* = .015, η_p_^2^ = .15 (and no main effects were significant, *p*s > .12, η_p_^2^ ≤ .06). Pairwise comparisons revealed that infants in the active and observational training conditions differed significantly in their attention to synchronous events, *md* = 5.31 (*SEM* = 1.83), *p* = .006 (*estimated marginal means* = 19.11 and 13.80, respectively), but not asynchronous events, *md* = .40 (*SEM* = 2.03), *p* = .85, in the 600 ms offset trials (see **Fig 4**).

Finally, analyses comparing the proportion of synchrony preferred trials for the active and observational conditions at each delay offset were conducted. That is, independent samples-tests were conducted in order to investigate whether the proportion of trials for which infants preferred looking to the synchronous videos differed between the two conditions. Consistent with the above-summarized analyses with raw looking times, this revealed a significant difference between conditions for the 600 ms delay offset, *t*(38) = 2.42, *p* = .02, but no significant difference for the 300 ms delay offset, *t*(38) = .14, *p* = .89. The difference between conditions in the 600 ms offset delay was driven by a difference from chance in relative synchrony preference in the active condition, *t*(19) = 3.68, *p* = .002, but not in the observational condition, *t*(19) = -.69, *p* = .50. Neither condition significantly differed from chance in the 300 ms offset trials, *t*s ≤ 1.0, *p*s > .30.

### Discussion

Infants who received similar observational experience to infants’ own observed actions in Experiment 1 (i.e., a similar number of perceivable drum hits) showed no evidence of sensitivity to multisensory synchrony in this paradigm. The three-way-interaction between Synchrony, Delay Offset, and Condition when comparing the raw looking times from the active condition from Experiment 1 and the observational condition in Experiment 2 suggests that observational experience was not as effective as active experience for detecting multisensory synchrony when the asynchronous visual and auditory information was offset by 600 ms. The data concerning the consistency of synchrony preference (i.e., the proportion score) indicated that there was a difference between conditions in relative preference for the synchronous events at the 600 ms, but not 300 ms, offset. During 600 ms offset trials, infants in the active condition differed from chance in their preference for synchronous events, but infants in the observational condition did not. The specificity of the effect for this longer offset (and the lack of overall effect for the 300 ms offset) is in accord with findings suggesting that greater offsets are recognized as asynchronous earlier in life and that behavioral effects for latencies of less than 400 ms are rare in the first half year of life [6, 8].

The fact that observational experience was not effective in facilitating multisensory integration in this experimental context, however, does not imply that it does not play a role in the development of sensitivity to multisensory integration at all. The experience infants in this study received, though matched to that of the observational experience infants in the active training condition from Experiment 1 received, was quite minimal. It is possible that more extensive observational experience or different kinds of observational experience would be more effective. The current findings suggest though, that with minimal experience, active experience is more beneficial than observational experience, for differentiating synchronous and (600 ms offset) asynchronous stimuli.

## General Discussion

Together, these experiments suggest that active experience provides a unique benefit, above and beyond observational experience, for sensitivity to multisensory synchrony (i.e., differentiation between synchrony and asynchrony) in the first year of life. Importantly, the observational condition accounted for the average number of times infants saw and heard the actions being produced, thus diminishing the possibility that sheer familiarity with the multisensory consequences of the action drove any effects in the active condition. This research was conducted with six-month-old infants, an age at which infants are at the brink of performing two-step actions such as drum beats and are beginning to differentiate synchronous versus asynchronous multisensory presentations of audiovisually presented events [8]. The results are thus consistent with previous research demonstrating an effect of active experience on perception of events in a variety of domains at the cusp of action production (e.g., understanding of means-end goals [42] and understanding of mental rotation [43]).

An important question concerns the mechanism underlying the benefits of active experience. That is, why should active experience producing multisensory effects lead to better recognition of the integration of these effects when later perceiving these effects? A low-level explanation for the difference in the effect might be an effect of general attentional differences between conditions. In Experiment 1, infants in the active training and control conditions differed in their overall attention to the video events in this experiment. Although effects of active training, relative to no training, were consistent when accounting for differential overall attention, it is possible that different engagement levels could have influenced infants’ differential attention during test trials. In order to avoid this possibility in future research, one possibility would be to train infants in the control condition on an alternative action (one unrelated to that being tested). In the current research, however, the observational training in Experiment 2 controlled for any possible effect of attentional differences. There were no differences in overall attention to the video events between infants in the active and observational training conditions. Further, data comparing attention to the events during active versus observational training suggest that no differences in attention to the drumming events during training could drive the observed effects. That is, infants in the observational condition heard just as many drum hits as infants in the active training condition and they actually watched as many or more of these drum hits than infants in the active training condition. Thus, no differences in attention (as assessed via gaze and/or possibility of hearing) to the event prior to the video session could have driven infants’ sensitivity to the synchrony of the observed event.

An alternative possibility is that, having carried out the action oneself, one can better predict the timing of the consequences of perceived actions. This hypothesis is in accord with research discussed in the introduction concerning the role of active experience in predictions about perceived actions (e.g. [28]). Lee and Noppeney [31] hypothesized that differences in multisensory binding between piano players and novices was due to the predictive ability of the musicians: “Because piano playing generates sensory signals in multiple modalities, the internal forward model indirectly also furnishes predictions about the relative timings of the auditory and visual signals leading to a narrower temporal binding window (p. E1441).” They found both behavioral and neural effects of experience in this research, but it was conducted with adults who had years of experience piano playing (see also [44, 45]) and the experience was not randomly assigned, thus leaving several confounds uncontrolled. To more clearly test the predictive hypothesis put forth by these authors, they suggested that future studies should directly contrast effects of pure audiovisual vs. audiovisual-motor training on audiovisual synchrony perception. The current study is a clear example of this comparison. Although consistent with this possibility, the present findings do not, however, directly test the consequences of active versus passive experience on prediction. Recent research with developmental populations using eye-tracking to measure infants’ predictive eye movements during perception of goal-directed movements indicates that active experience influences infants’ predictive encoding of others’ behavior (e.g. [46]). Consistent with the current results, research with eight-month-old infants that contrasted short-term active versus observational training with particular actions found unique effects of active experience on prediction during subsequent observation of the action [47]. These findings are consistent with the current research in indicating that the forward model is a probable mechanism of both action prediction and, in this case, sensitivity to multisensory synchrony.

The depth and breadth of the effects found in the active training condition are currently undefined. For example, how long-lasting are the effects of the active training experience? It seems unlikely that a short, five-minute training session would lead to a permanent change in sensitivity, but the amount of experience needed over time and the duration of the effect given different periods of experience could be explored in future studies. Another open question concerns the specificity of the effects observed in this study. Whether training has an effect on sensitivity to audiovisual synchrony across tasks and events or is specific to the particular action learned was not addressed in the present study. The specificity or generalization of this learning is an important topic for further investigation

By highlighting the crucial role of active experience for infants’ cognition and perception, the present study is relevant to the field of early musical development. Indeed, while many contributions in this area suggest that infants display sensitivity for certain musical features (for example consonance and dissonance) without formal training [48] and that simple exposure is sufficient to the development of highly specialised brain circuits devoted to process Western music [49–51], our study shows that sensorimotor activity consolidates the sensitivity to audiovisual synchrony, promoting a more *embodied* view of the processes involved in learning [52]. Although we do recognize the importance of environmentally rich information for the development of one’s musical abilities [23], we also argue that infants are active creators of experience from birth [53], and thus cannot be considered passive perceivers of stimuli, and are rather active and dynamic participants in shaping their environment [54–58]. Our contribution, in this sense, aligns with the growing number of studies that recently highlighted the role of active training for music skill acquisition [59–61]. In general, this perspective on musical learning as grounded in sensorimotor activity is consistent with the influential work by Esther Thelen, who explored the dynamics of human development without positing any explicit dichotomy between categories such as action, learning, cognition and development (e.g. [62–63]). We believe that integrating this framework within the sphere of infants’ musicality will stimulate further research to improve our understanding of musical development in infancy.

Given the role of musical expertise on sensitivity to audiovisual synchrony, one might expect that the quality of infants’ training experience might influence infants’ relative sensitivity. Although we assessed individual differences in the number of drumming actions produced by infants and in their attention (i.e., gaze) to these actions and their effects, these analyses were not reported in the manuscript because we found no relation between individual differences in production or attention to these actions during training and infants’ differentiation between synchrony and asynchrony in the video session. Thus, quality of training, as measured via amount of produced actions (both by self and/or with the help of an adult) and relative attention to the produced actions, was unrelated to sensitivity to multisensory synchronization in the current experiment. This null effect does not necessarily suggest that quality of training is uninformative more generally. It is possible that, with increased training periods or more fine-grained measures of sensitivity to multisensory synchronicity, effects of training quality could be revealed in subsequent experiments.

Future research should examine the relative benefits of active versus observational experience on sensitivity to multisensory synchrony over time. For example, the specific difference between active and observational training at a 600 ms, rather than a 300 ms, delay is likely a function of the young age of the participants but can be directly assessed in future work by examining this effect throughout development. Further, future studies can explore whether observational experience across an extended period of time or later in development is as effective as active experience. Finally, an important avenue of future research concerns the limitations or conjunctive effects of general brain maturation on the experience infants receive producing and perceiving multisensory actions. The current findings provide insight into the underlying mechanisms of sensitivity to audiovisual synchrony. They suggest that playing an active role in creating multisensory effects is uniquely beneficial for perception of the synchrony of similar audiovisual stimuli. This finding could have implications for the role of different kinds of experience on infants’ perception of the world in a variety of domains that require multisensory processing, including speech perception, action understanding, and musical cognition.

## Supporting Information

S1 File Complete dataset. An excel document includes all data used in the analyses reported in the Results section.
(XLSX)
